# Conservative treatment of gastric perforation after microwave ablation of a hepatocellular carcinoma: Case report

**DOI:** 10.1097/MD.0000000000029195

**Published:** 2022-06-03

**Authors:** J. Roller, V. Zimmer, A. Bücker, M. Glanemann, R.M. Eisele

**Affiliations:** a Department for General-, Visceral-, Vascular and Pediatric Surgery, University Hospital of the Saarland, Homburg, Saar, Germany; b Department for Internal Medicine, Marienkrankenhaus St. Joseph, Neunkirchen, Germany; c Department for Diagnostic and Interventional Radiology, University Hospital of the Saarland, Homburg, Saar, Germany; d Surgical Center Oranienburg, Oranienburg, Germany.

**Keywords:** complication, gastric perforation, hepatocellular carcinoma, liver transplantation, microwave ablation, radiofrequency ablation

## Abstract

**Rationale:**

Microwave ablation (MWA) has been proven to be an efficient and safe method for local tumor control of liver tumors. Reported complications are rare, but include liver abscess, hematoma, pleural effusion, and occasional thermal injury of the adjacent colon. Intestinal perforation usually requires immediate surgical treatment to prevent generalized peritonitis and sepsis.

**Patient concerns and diagnosis:**

Herein, we describe a case of gastric perforation following percutaneous MWA for hepatocellular carcinoma as a bridging therapy prior to liver transplantation.

**Interventions:**

Due to the clinical condition of the patient, conservative treatment was considered sufficient. Nine months after MWA, successful liver transplantation followed. Intraoperative findings revealed a scar in the gastric wall with tight adhesions to the liver, requiring adhesiolysis and subsequent suturing. Postoperative recovery was uneventful.

**Outcome:**

At present, the patient is doing well. No further gastrointestinal events occurred.

**Lesson:**

To our knowledge, this is the first report of such a complication occurring after MWA. Moreover, in this case, the gastric perforation could be treated conservatively.

## 1 Introduction

Hepatocellular carcinoma (HCC) is one of the leading causes of cancer-related deaths worldwide.^[1]^ The gold standard for the treatment of HCC is surgical tumor resection. However, a large number of patients do not qualify for open surgery because of multifocal tumor appearance in the liver or marginal hepatic function.^[2]^ These patients may benefit from local tumor control using locoregional treatment modalities. Microwave ablation (MWA) belongs to various treatment modalities that have been introduced in recent years. In this technique, high-frequency electromagnetic energy is applied to the center of the tumor using a special applicator. In doing so, the tissue near the antenna can be heated to cytotoxic levels.

MWA has been proven to be an efficient and safe method.^[3]^ However, due to the application of heat at the cytotoxic level to the target tissue, distinct complications may evolve. Heat injury to the gastrointestinal walls after MWA has been described previously.^[4]^

Usually, transmural thermal injury of the gastrointestinal wall requires surgical treatment. However, with covered perforations and a lack of signs of peritonitis or systemic inflammation, conservative treatment can occasionally be considered as an exception.

Herein, we describe a case of gastric perforation following MWA of a HCC nodule. Interestingly, this complication was treated conservatively in this case.

## 2 Case report

A 62-year old patient with cirrhosis due to chronic hepatitis C infection presented with bifocal HCC recurrence. Open atypical resection of a HCC in segment VII was performed 4 years previously. The HCC nodules were now located in segments III and VIII. Due to the impaired general condition and poor liver function, MWA of the HCC-nodules was scheduled as bridging therapy prior to liver transplantation. Based on the accessibility of the tumor nodes and minimal invasiveness, a percutaneous approach was chosen. The intraoperative course was uneventful with a total procedure time of 48 minutes. MWA was performed under sonographic guidance for 4 minutes (segment III) and 5 minutes (segment VIII) using a 14 gauge probe (MedWaves Avecure^TM^ Probe and Microwave Generator; MedWaves Inc., San Diego, CA). At the end of the procedure, ultrasonography revealed complete ablation of the nodules. Radiographic imaging of the abdomen could exclude the presence of free abdominal air after MWA.

Postoperatively, the patient had a regular postoperative course. In line with this, inflammation parameters were only slightly increased postoperatively (maximum of C-reactive protein at day 5 after MWA: 95.6 mg/L; reference value: 0–5 mg/L) with no increased systemic leukocyte counts.

**Figure 1:**
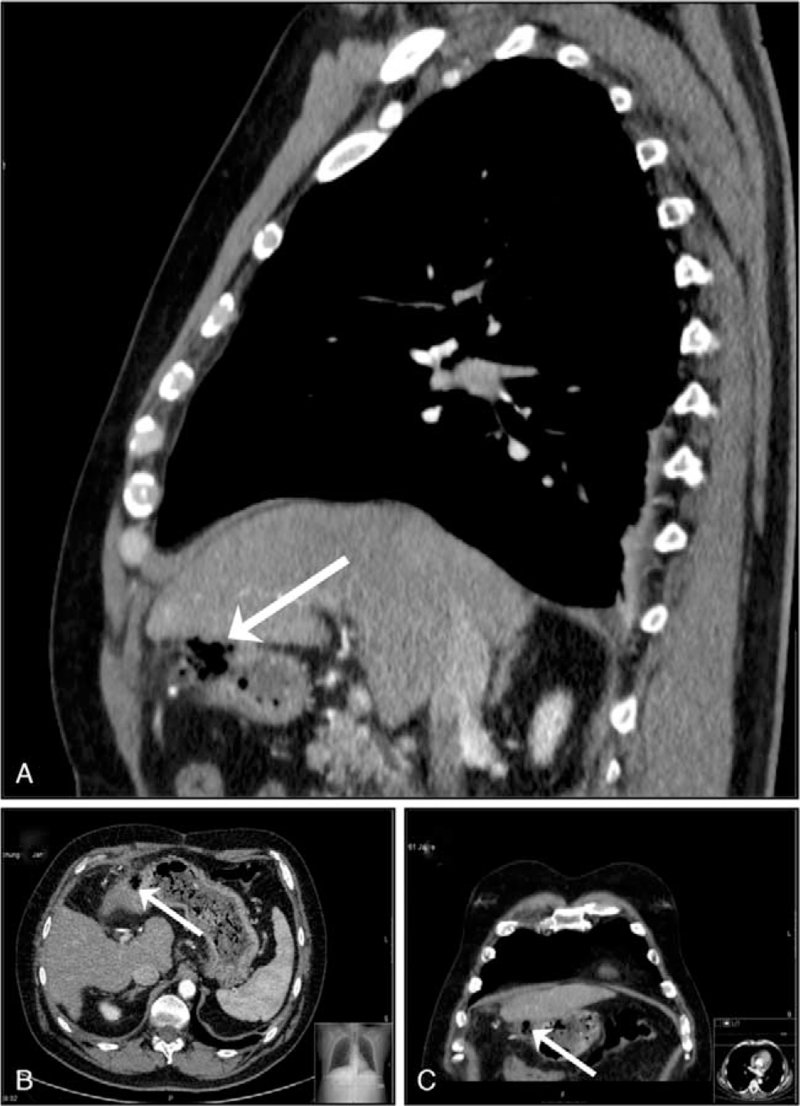
Contrast enhanced computed tomography 2 weeks after percutaneous microwave ablation of hepatocellular carcinoma nodules in segments III and VIII. Sagittal and horizontal images reveal a defect of the gastric antrum wall in direct vicinity a necrotic ablation area in segment III of the liver (A, B, arrows). Of interest, frontal images show that the adjacent liver covers the defect (C, arrow).

Two weeks after MWA, computed tomography (CT) angiography was performed for further evaluation of liver transplantation. Herein, a defect of the gastric antrum wall in direct vicinity to the necrotic ablation area in segment III of the liver was discerned. The defect presented with a size of 1.2 cm and gas inclusions (Fig. 1, arrows). However, no intraperitoneal gas or liquid was observed, as the adjacent liver covered the defect. Due to the absence of signs of systemic inflammation, peritonitis, and good clinical condition of the patient, conservative treatment with proton pump inhibitors and systemic antibiotics was decided.

**Figure 2:**
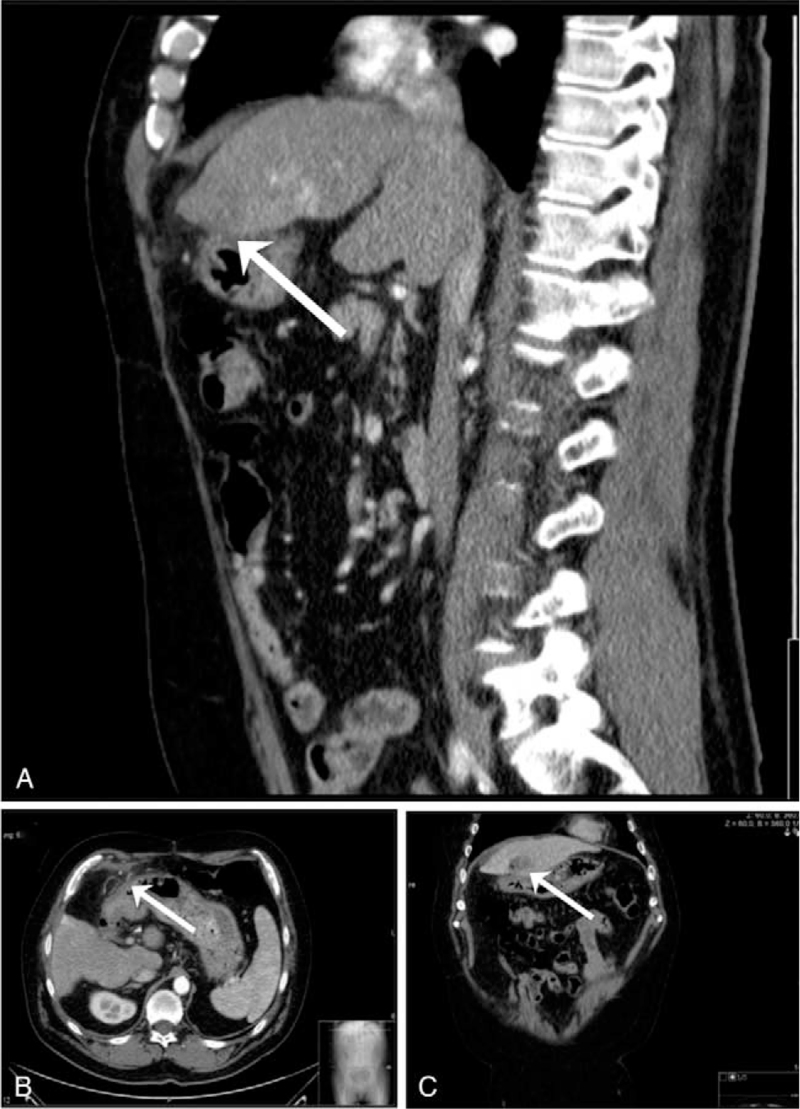
Contrast enhanced computed tomography 6 weeks after percutaneous microwave ablation of hepatocellular carcinoma nodules in segments III and VIII. Sagittal and horizontal images reveal the integrity of the gastric wall in the area of the former perforation (A, B, arrows). Frontal images show the intact gastric wall in direct vicinity to the necrotic ablation area (C, arrow).

**Figure 3:**
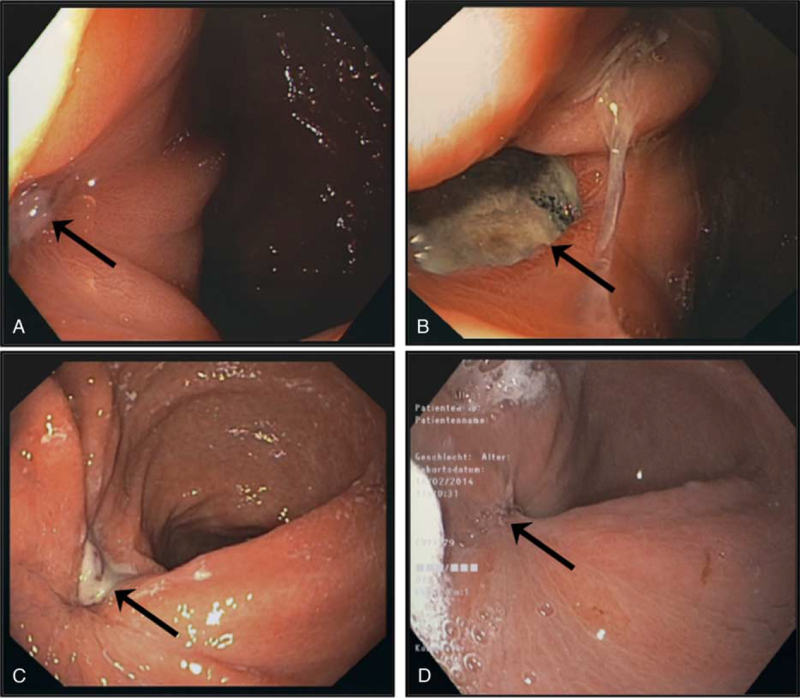
Gastroscopy 6 weeks after microwave ablation of a hepatocellular carcinoma nodule revealed an ulcer of the antrum wall with substantial scarred retraction of the gastric wall in this area (A). Gastroscopic controls were performed 2 (B), 3 (C), and 4 (D) months after microwave ablation. After 4 months (D), the defect had completely disappeared following conservative treatment.

After 6 weeks, a CT scan of the abdomen did not show a defect in the gastric wall near the ablation area in segment III (Fig. 2, arrows). Gastroscopy revealed an ulcer located in the antrum, with substantial scarred retraction of the gastric wall in this area (Fig. 3A). Interestingly, this ulcer had not been observed in prior gastroscopy, and the location correlated well with the defect of the gastric wall after the initial MWA. Repeated gastroscopy 2 months after ablation and after 3 and 4 months revealed further healing of the gastric wall (Fig. 3B, C, D). Liver transplantation was performed 9 months after ablation. Currently, the patient is doing well with good liver function in outpatient care after liver transplantation.

## 3 Discussion

Ablative treatment of liver tumors represents the option of choice for local tumor control in patients who do not qualify for open resection.^[5]^ Beyond palliative treatment of surgically non-resectable tumors, ablation of HCC nodules can be used as bridging therapy before liver transplantation. In recent years, several treatment modalities for ablation of tumors in the liver have evolved. To date, thermo-ablative methods such as radiofrequency ablation (RFA) and MWA have been favored in most centers. It has been shown that RFA provides significantly increased survival rates compared to ethanol instillation into tumors.^[6]^ Moreover, the complication rate was significantly lower.^[7]^ In comparison to RFA, MWA provides technical advantages, such as a larger zone of coagulation necrosis and higher temperatures within the tissue, leading to shorter treatment duration and fewer heat-sink effects.^[6]^ Owing to the high temperature and larger ablation areas in the case of MWA, safety concerns have appeared. In fact, in our case, it was assumed that the defect within the gastric wall was caused by thermal injury. However, it is important to note that it has been shown that complications of MWA do not differ from those of RFA,^[8]^ and it is not likely that gastric perforation might have been avoided by the application of a different thermoablative treatment modality (e.g., RFA).

Thermoablation of liver tumors can be performed using open surgical, laparoscopic, or percutaneous approaches. Laparoscopic and open access have the advantage of increased tumor control compared with percutaneous ablation.^[9]^ Laparoscopic ablation offers specific technical advantages, as the positive pressure of the pneumoperitoneum decreases portal venous blood flow and thus increases the ablation area.^[10,11]^ Moreover, the percutaneous approach is associated with an increased risk of perforation of the gastrointestinal wall due to thermal injuries. This risk is especially present when treating lesions adjacent to the gastrointestinal lumen and in patients with a history of abdominal surgery, intestinal adhesions, or anatomical variations.^[12]^ However, in patients with HCC due to liver cirrhosis, an open surgical or laparoscopic approach may not be favored because of their poor clinical condition. Accordingly, bridging patients with HCC for orthotopic liver transplantation is performed percutaneously in most cases. In our patient, a percutaneous approach was chosen, although an open atypical liver resection of an HCC had previously been performed. Causes for this preference were poor hepatic function and planned liver transplantation as a definitive treatment for HCC.

MWA for HCC is associated with a low complication rate, ranging from 0% to 11%.^[13,14]^ Reported complications include thermal injury of the skin, liver hematoma and abscess, pneumothorax and pleural effusion.^[9,15]^ Moreover, thermal injury to the colon has been described previously.^[4]^ To the best of our knowledge, no gastric perforation following MWA of HCC has been described to date. In fact, practical experience with other thermo-ablative treatment modalities (e.g., RFA) shows that gastric perforation due to thermal injury is a rare entity compared to colonic perforation. This may, at least in part, be caused by the relative thickness of the gastric wall compared to that of the colon.^[16]^

Interestingly, the described intestinal perforations did not occur immediately after ablation but on days 3 to 5 after MWA. This time course might be due to the fact that MWA causes a coagulation necrosis of the tissue. Secondary perforation became apparent after decomposition of the necrotic tissue. Unfortunately, in our case, we could not determine the exact time point of perforation, as the patient presented with a regular postoperative clinical course, and the perforation was diagnosed on a CT-scan 2 weeks after MWA. However, retrospectively, the peak in the serum inflammatory values (C-reactive protein) could be noticed 5 days after ablation and may have been a correlate of gastric perforation.

Intestinal perforations usually require immediate surgical treatment. However, several cases have been described where gastric perforation at early diagnosis could be treated endoscopically and emergency surgery could be avoided.^[17,18]^ In individual cases with localized peritonitis and a clinically stable condition, conservative treatment of the perforation might be successful.^[19]^ In the described case, the patient did not present with any clinical signs of gastric perforation. The perforation was incidentally diagnosed 2 weeks after ablation. By that time, regressing inflammation parameters had been observed, and the patient had presented with no signs of peritonitis. MWA was performed as bridging therapy for HCC before liver transplantation. In addition, the patient presented with poor liver function due to cirrhosis. To avoid a delay in planned transplantation arising from an explorative laparotomy and suturing of the gastric perforation, further conservative treatment was decided. Under close gastroscopic monitoring, healing of the gastric perforation was observed, and liver transplantation was successfully performed 9 months after MWA.

## Author contributions

**Conceptualization:** Robert Martin Eisele.

**Data curation:** Vincent Zimmer.

**Formal analysis:** Jonas Roller.

**Funding acquisition:** Robert Martin Eisele.

**Methodology:** Matthias Glanemann, Robert Martin Eisele.

**Resources:** Vincent Zimmer, Arno Buecker.

**Supervision:** Matthias Glanemann.

**Validation:** Robert Martin Eisele.

**Visualization:** Arno Buecker.

**Writing – original draft:** Jonas Roller.

**Writing – review & editing:** Robert Martin Eisele.
